# How to predict the electronic health literacy of Chinese primary and secondary school students?: establishment of a model and web nomograms

**DOI:** 10.1186/s12889-022-13421-4

**Published:** 2022-05-25

**Authors:** Tao Xie, Ning Zhang, Ying Mao, Bin Zhu

**Affiliations:** 1 School of Public Policy and Administration, Xi’an Jiaotong University, 28 Xianning West Road, Xi’an 710049, Beilin District China; 2 School of Public Health and Emergency Management, Southern University of Science and Technology, Shenzhen 518055, Guangdong China

**Keywords:** Electronic health literacy, Chinese students, Random forest, Lasso, Web nomograms

## Abstract

**Background:**

The internet has become an important resource for the public to obtain health information. Therefore, the ability to obtain and use such resources has become important for health literacy. This study aimed to establish a prediction model of Chinese students’ electronic health literacy (EHL) to guide government policymaking and parental interventions, identify the predictors of EHL in Chinese students using random forests, and establish a corresponding prediction model to help policymakers and parents determine whether primary and secondary school students have high EHL.

**Methods:**

This is a cross-sectional study. From June to August 2021, a cluster sample survey was conducted with 1,300 students from seven primary and secondary schools in Shaanxi Province, China. We evaluated 1,235 primary and secondary school students using the e-health literacy scale. The data were divided into training and testing datasets in a 70:30 ratio for further analysis using random forest. The predictive accuracy of the score was measured using the area under the receiver operating characteristic curve. We also used decision curve analysis to determine the usefulness of the prediction model by quantifying the net benefits at different threshold probabilities in the validation dataset.

**Results:**

We found that 33.6% of students had high EHL. The univariate analysis showed that age (*P* < 0.001), grade (*P* < 0.001), employment status (*P* < 0.001), household location (*P* < 0.001), parental phubbing behavior (*P* < 0.001), and general self-efficacy (*P* < 0.001) were significantly associated with EHL. A random forest classification model was developed with the training dataset (872 students), and seven variables were confirmed as important: age, grade, employment status, father education level, game time, parental phubbing behavior, and general self-efficacy. The validation of the model showed good discrimination, with an area under the curve of 0.975 in the training dataset and 0.738 in the testing dataset. The model was translated into an online risk calculator, which is freely available (https://xietao.shinyapps.io/DynNomapp/).

**Conclusions:**

In this study, an intuitive tool to predict the EHL of Chinese primary and secondary school students was developed and validated.

**Supplementary Information:**

The online version contains supplementary material available at 10.1186/s12889-022-13421-4.

## Background

Health literacy refers to the ability of individuals to obtain and understand health information and make correct health decisions [1]. Electronic health literacy (EHL), first proposed by Canadian scholar Norman et al. [2], refers to the ability of individuals to obtain, understand, judge, and use information from electronic resources to solve their health problems. It is a concept that combines HL and electronic health [3]. The e-health literacy scale (eHEALS), prepared by Norman et al. [4], is the first and currently the most commonly used EHL assessment tool. It mainly measures the self-perception skills of internet users when they seek and apply online health knowledge.

With the rapid development of internet technology, an increasing number of government departments, medical institutions, and nonprofit organizations have placed health-related information on the internet. Many people have begun to obtain health information through the internet, and EHL is gaining attention [5, 6]. However, not everyone has the HL to access appropriate health information, especially primary and secondary school students.

The popularity of the internet is quite high among primary and middle school students who are familiar with the most popular network applications and rely on network information technology for all kinds of communication, interaction, and access to information related to life and learning. However, previous studies have shown that junior high school students are not able to make good judgments about online health information and cannot use the internet to help solve health problems [7]. Therefore, the ability to obtain and use such resources has become an important component of individual HL [8]. Middle school students are in a critical development period where their world outlook, life outlook, and values form, and their ability to distinguish between good and bad information on the internet is not mature [9].

However, Chinese schools in this group have low basic knowledge of electronic media and EHL. If this problem is ignored, it will not be conducive to the balanced and healthy development of these students [10]. In China, studies mainly focus on the current situation and influencing factors of EHL [11–16], the relationship between EHL and having a healthy lifestyle [17, 18], and the current situation of searching for health information on the internet [19]. For example, to understand the status of EHL among college students in Guangdong province during the COVID-19 pandemic, Pan Chenghao et al. [11] conducted an online questionnaire survey among college students in Guangdong province and found that the level of EHL was low and female students and those who were more affected by information related to COVID-19 had lower EHL. Liu Jianchao et al. [18] selected 1157 college students from four higher vocational colleges in Jinan to investigate EHL and disease behavior and found that EHL is an important factor that affects the disease behavior of college students in higher vocational colleges. The above studies mainly focused on college students, and there are few studies on EHL among primary and secondary school students [9, 10, 20]. Linan et al. [9] used the eHEALS scale to conduct an EHL survey of middle school students, and the results showed that adolescents had low application ability and evaluation ability in obtaining online health information and services. Xie Yuchang et al. [20] found that high school students have a certain level of EHL and interactive HL through a study of EHL in high school students and that the two were positively correlated.

Although some international studies have examined the factors of EHL in adolescents, most of the focus is on recognition and college students. For example, Holch et al. [21] found that eHEALS was significantly positively correlated with general self-efficacy and that general self-efficacy was a significant predictor of eHEALS scores. Amina Tariq et al. [22] showed that perceived EHL was not associated with health behaviors such as physical activity and dietary supplement intake. Adile et al. [23] indicated that the mean digital HL scores were high in students who lived in a nuclear family, understood the importance of good health, had easy access to the internet, and had highly educated parents with high-income levels in Turkey. Tsukahara et al. [24] reported that the EHL of university students in Japan was comparable to that of the general Japanese population. Graduate students, as well as those in medical departments, had higher EHL. It appears from the above studies that EHL is related to socio-demographic and socio-economic variables.

Unfortunately, no specific studies have predicted EHL among Chinese primary and secondary school students. Therefore, identifying and predicting the EHL of primary and secondary school students is critical. This study aimed to identify the predictors of EHL in Chinese students using random forest and establish a corresponding prediction model to help policymakers and parents determine whether primary and secondary school students have EHL to enable them to implement more targeted interventions.

## Methods

### Study type

This study was designed as a cross-sectional study.

### Study design and data collection

A total of 1300 students from seven primary and middle schools in Shaanxi Province, China, were surveyed from June to August 2021. In this study, cluster sampling was used to randomly select two primary schools, two middle schools, and three high schools in the main urban areas of Yulin City and Ankang City of Shaanxi Province. Four classes were randomly selected from each primary school, and four classes were randomly selected from each middle school and high school. The inclusion criteria were public schools, elementary students in grades 2–5, middle school students in grades 1–2, and high school students in grades 1–2. The exclusion criteria included private schools, first and sixth graders, junior middle school students, and senior high school students.

Two to four researchers were responsible for each study. To ensure the quality of the questionnaire, the students were guided by the researchers during the questionnaire-filling process. After explaining our study, informed consent was obtained from all participants or their legal guardians for those below 16 years old. Of the 1300 students interviewed, 65 were excluded from the analysis because of the large number of missing values in the questionnaire. We then randomly divided them into training and testing datasets at a ratio of 70:30, with 872 students assigned to the training database and 363 students assigned to the testing database.

All methods were performed in accordance with the transparent reporting of a multivariable prediction model for individual prognosis or diagnosis (TRIPOD) [25] guideline and regulation.

### Potential predictive variables

We conducted a systematic review of HL in Chinese students [26], identifying all published observational studies in both Chinese (CNKI, Wan Fang, CQVIP) and English databases (PubMed, Embase, Web of Science, Cochrane Library) between January 2010 and September 2020 on factors that affect HL in Chinese students. The significant influencing factors for Chinese students were sex, location of the household grade, good academic performance, race, health information concerns, online game time, parental education, whether they were a single child, family monthly income, health education, if they were majoring in medicine or attending medical school. Therefore, we identified the following potential predictive variables for this study: sex, age, race, grade, family size, only child, employment status, household location, mother’s education, father’s education, and gaming time. We did not consider health information concerns, majoring in medicine, and medical school attendance because the influence group of these variables is college students in the systematic review. Academic performance was not included in the analysis because China’s current policy regards student performance as very important and private and is therefore difficult to obtain in the data collection process. Most primary and middle school students do not know their family income. Therefore, we did not include family income in the analysis. In addition to the factors mentioned above, another study found that self-efficacy and parental phubbing behavior were closely related to HL [27, 28]. Therefore, these two variables were included. General self-efficacy was measured using the general self-efficacy scale (GSES) [29], and parental phubbing behavior was measured using the parental phubbing scale (PPS) [30].

### Outcomes

We used the eHEALS prepared by Norman et al. [2] to evaluate the EHL of primary and secondary school students (with vs. without). Students who scored above 80% were judged to have EHL [31]. We used 80% of the scoring nodes because we borrowed the Chinese HL classification method. There have also been other studies [15, 32] that have determined EHL using the 80% threshold. See Additional file 1 for more detailed information and the reliability and validity analysis of the scale.

### Statistical analysis

Bivariate analysis was performed using the Mann–Whitney U test for continuous and ordinally distributed variables and the chi-squared test for categorical variables. For further analysis, a nomogram was formulated based on the machine learning results.

Random forest, a classical algorithm in machine learning, was selected for learning and prediction. The basis of random forest is a decision tree, which is a basic classification and regression method. The decision tree model takes the form of a tree. A classification problem represents the process of classifying instances based on their features. Random forest is an algorithm that combines the results of multiple decision trees for classification or regression. The number of decision trees constructed in this study was 500, and three variables were randomly selected for each node of the decision tree. Random forests select or exclude variables based on the importance of the features. Validated variables were used to create a simplified model rather than a complete model with all variables. Similar to other machine learning models, the random forest algorithm consists of training and testing steps. The computer first uses a training set to select the optimal model and then uses a test set to evaluate the model. The area under the curve (AUC) was used as an assessment tool, and AUC values between 0.6 and 0.8 were considered acceptable [33].

The least absolute shrinkage and selection operator (LASSO) is a regression analysis method used for simultaneous feature selection and regularization. This adds an L1 norm as a penalty in the calculation of the minimum residual sum of squares. When lambda is sufficiently large, certain coefficients can be accurately reduced to zero. LASSO has excellent feature selection ability. Therefore, we also conducted LASSO regression and compared the results with random forest.

The receiver operating characteristic (ROC) curve is drawn on a two-dimensional plane. It was drawn with sensitivity as the ordinate and specificity as the abscissa. Any point on the curve represents the corresponding sensitivity and specificity for the observed sample. The AUC refers to the size of a part of the area under the ROC curve, which is a standard used to measure the quality of a classification model and reflects the accuracy of the model. Typically, AUC values range from 0.5 to 1.0, with a larger AUC representing better model performance.

Decision curve analysis (DCA) reflects outcome variables and can be used to evaluate and compare different prediction models. The AUC only measures the accuracy of the prediction model and does not consider the actual utility of a particular model, whereas the DCA integrates the preferences of the object or decision-maker into the analysis.

To facilitate the application of the prediction model, we developed a web page based on a prediction model using Shinayapp. Statistical analysis was performed using R version 4.0.5 for Mac (R Foundation for Statistical Computing).

## Results

### Characteristics of sample

We found that 415 (33.6%) students were e-health literate, and 820 (66.4%) were not. Table 1 summarizes the characteristics of the total population of Chinese students (*N* = 1235). The univariate analysis showed that age (*P* < 0.001), grade (*P* < 0.001), employment status (*P* < 0.001), household location (*P* < 0.001), parental phubbing behavior (*P* < 0.001), and general self-efficacy (*P* < 0.001) were significantly associated with EHL.

**Table 1:** Overview of the characteristics of all the datasets

Factor	Testing = 0 (*N* = 363)	Training = 1 (*N* = 872)	EHL = 0 (*N* = 820)	EHL = 1 (*N* = 415)	*p*-value
Sex					0.81
Male	188 (51.8%)	423 (48.5%)	408 (49.8%)	203 (48.9%)	
Female	175 (48.2%)	449 (51.5%)	412 (50.2%)	212 (51.1%)	
Age	14.0 (11.0, 17.0)	14.0 (11.0, 17.0)	14.0 (11.0, 17.0)	16.0 (13.0, 17.0)	< 0.01
Race					0.41
Han	360 (99.2%)	861 (98.7%)	809 (98.7%)	412 (99.3%)	
Nhan	3 (0.8%)	11 (1.3%)	11 (1.3%)	3 (0.7%)	
Grade					< 0.01
2	105 (28.9%)	243 (27.9%)	259 (31.6%)	89 (21.4%)	
3	102 (28.1%)	246 (28.2%)	234 (28.5%)	114 (27.5%)	
4	152 (41.9%)	375 (43.0%)	318 (38.8%)	209 (50.4%)	
5	0 (0.0%)	1 (0.1%)	1 (0.1%)	0 (0.0%)	
7	2 (0.6%)	7 (0.8%)	7 (0.9%)	2 (0.5%)	
8	2 (0.6%)	0 (0.0%)	1 (0.1%)	1 (0.2%)	
Family size	4.0 (4.0, 4.0)	4.0 (4.0, 4.0)	4.0 (4.0, 4.0)	4.0 (4.0, 4.0)	0.81
Only child					0.84
Yes	29 (8.0%)	99 (11.4%)	84 (10.2%)	44 (10.6%)	
No	334 (92.0%)	773 (88.6%)	736 (89.8%)	371 (89.4%)	
Employment status					< 0.01
Father working	97 (26.7%)	243 (27.9%)	244 (29.8%)	96 (23.1%)	
Mother working	6 (1.7%)	27 (3.1%)	21 (2.6%)	12 (2.9%)	
Parents both working	42 (11.6%)	109 (12.5%)	113 (13.8%)	38 (9.2%)	
No parent working	218 (60.1%)	493 (56.5%)	442 (53.9%)	269 (64.8%)	
Location of the household					< 0.01
City	138 (38.0%)	312 (35.8%)	324 (39.5%)	126 (30.4%)	
Rural	225 (62.0%)	560 (64.2%)	496 (60.5%)	289 (69.6%)	
Mother education	2.0 (1.0, 3.0)	2.0 (1.0, 3.0)	2.0 (1.0, 3.0)	2.0 (1.0, 3.0)	0.07
Father education	2.0 (2.0, 3.0)	2.0 (2.0, 3.0)	2.0 (2.0, 3.0)	2.0 (2.0, 4.0)	0.55
Game time of online	2.0 (1.0, 3.0)	2.0 (0.0, 3.0)	2.0 (0.0, 3.0)	2.0 (1.0, 3.0)	0.91
Parental phubbing behavior	22.0 (17.0, 27.0)	22.0 (17.0, 27.0)	21.0 (17.0, 26.0)	24.0 (18.0, 28.0)	< 0.01
General self-efficacy	24.0 (20.0, 28.0)	24.0 (20.0, 28.0)	23.0 (20.0, 26.0)	27.0 (23.0, 31.0)	< 0.01

Table 2 summarizes the characteristics of the Chinese students in the training dataset (*n* = 872). Moreover, we found that 287 (32.9%) students were e-health literate, and 585 (67.1%) were not. The univariate analysis showed that age (*P* < 0.001), grade (*P* < 0.001), household location (*P* < 0.001), parental phubbing behavior (*P* < 0.001), and general self-efficacy (*P* < 0.001) were significantly associated with EHL.

**Table 2:** Overview of the characteristics of the training dataset

Factor	EHL = 0	EHL = 1	*p*-value
N	585	287	
Sex			0.89
Male	285 (48.7%)	138 (48.1%)	
Female	300 (51.3%)	149 (51.9%)	
Age	14.0 (11.0, 17.0)	16.0 (13.0, 17.0)	< 0.01
Race			0.52
Han	576 (98.5%)	285 (99.3%)	
Nhan	9 (1.5%)	2 (0.7%)	
Grade			< 0.01
2	190 (32.5%)	53 (18.5%)	
3	166 (28.4%)	80 (27.9%)	
4	223 (38.1%)	152 (53.0%)	
5	1 (0.2%)	0 (0.0%)	
7	5 (0.9%)	2 (0.7%)	
Family size	4.0 (4.0, 4.0)	4.0 (4.0, 4.0)	0.44
Only child			1.00
Yes	67 (11.5%)	32 (11.1%)	
No	518 (88.5%)	255 (88.9%)	
Employment status			0.05
Father working	172 (29.4%)	71 (24.7%)	
Mother working	18 (3.1%)	9 (3.1%)	
Parents both working	82 (14.0%)	27 (9.4%)	
No parent working	313 (53.5%)	180 (62.7%)	
Location of the household			< 0.01
City	233 (39.8%)	79 (27.5%)	
Rural	352 (60.2%)	208 (72.5%)	
Mother education	2.0 (1.0, 3.0)	2.0 (1.0, 3.0)	0.04
Father education	2.0 (2.0, 3.0)	2.0 (2.0, 4.0)	0.76
Game time of online	2.0 (0.0, 3.0)	2.0 (1.0, 3.0)	0.33
Parental phubbing behavior	21.0 (17.0, 25.0)	24.0 (19.0, 28.0)	< 0.01
General self-efficacy	23.0 (20.0, 26.0)	27.0 (23.0, 31.0)	< 0.01

Table 3 summarizes the characteristics of the Chinese students included in the testing dataset (*n* = 363). In the testing dataset, 128 (35.3%) students were e-health literate, and 235 (64.7%) were not. The univariate analysis showed that general self-efficacy (*P* < 0.001) was related to EHL.

**Table 3:** Overview of the characteristics of the testing dataset

Factor	EHL = 0	EHL = 1	*p*-value
N	235	128	
Sex			0.83
Male	123 (52.3%)	65 (50.8%)	
Female	112 (47.7%)	63 (49.2%)	
Age	14.0 (10.0, 17.0)	15.0 (11.0, 16.0)	0.73
Race			1.00
Han	233 (99.1%)	127 (99.2%)	
Nhan	2 (0.9%)	1 (0.8%)	
Grade			0.83
2	69 (29.4%)	36 (28.1%)	
3	68 (28.9%)	34 (26.6%)	
4	95 (40.4%)	57 (44.5%)	
7	2 (0.9%)	0 (0.0%)	
8	1 (0.4%)	1 (0.8%)	
Family size	4.0 (4.0, 4.0)	4.0 (4.0, 4.0)	0.47
Only child			0.54
Yes	17 (7.2%)	12 (9.4%)	
No	218 (92.8%)	116 (90.6%)	
Employment status			0.02
Father working	72 (30.6%)	25 (19.5%)	
Mother working	3 (1.3%)	3 (2.3%)	
Parents working	31 (13.2%)	11 (8.6%)	
No parent working	129 (54.9%)	89 (69.5%)	
Location of the household			0.74
City	91 (38.7%)	47 (36.7%)	
Rural	144 (61.3%)	81 (63.3%)	
Mother education	2.0 (1.0, 3.0)	2.0 (1.0, 3.0)	0.89
Father education	2.0 (2.0, 3.0)	2.0 (2.0, 4.0)	0.11
Game of online	2.0 (1.0, 3.0)	1.0 (0.0, 2.5)	0.19
Parental phubbing behavior	22.0 (17.0, 27.0)	23.5 (17.0, 28.0)	0.38
General self-efficacy	23.0 (20.0, 26.0)	27.5 (22.0, 31.0)	< 0.01

### Predictive variables selection

**Fig. 1:**
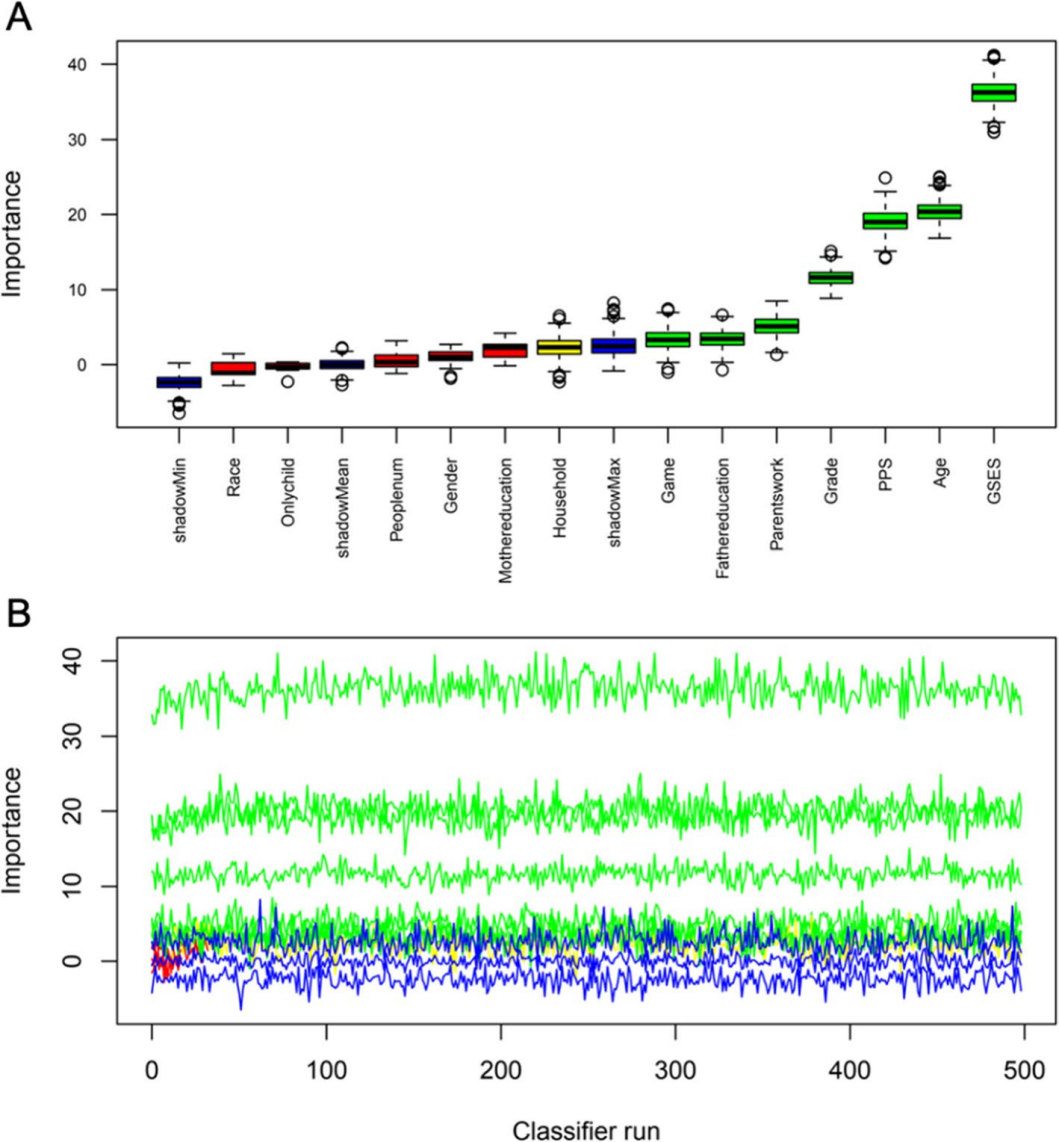
Feature selection and ranking by random forest. **A** The plot shows a boxplot of all variables. **B** History of decisions of rejecting or accepting features by random forest

**Fig. 2:**
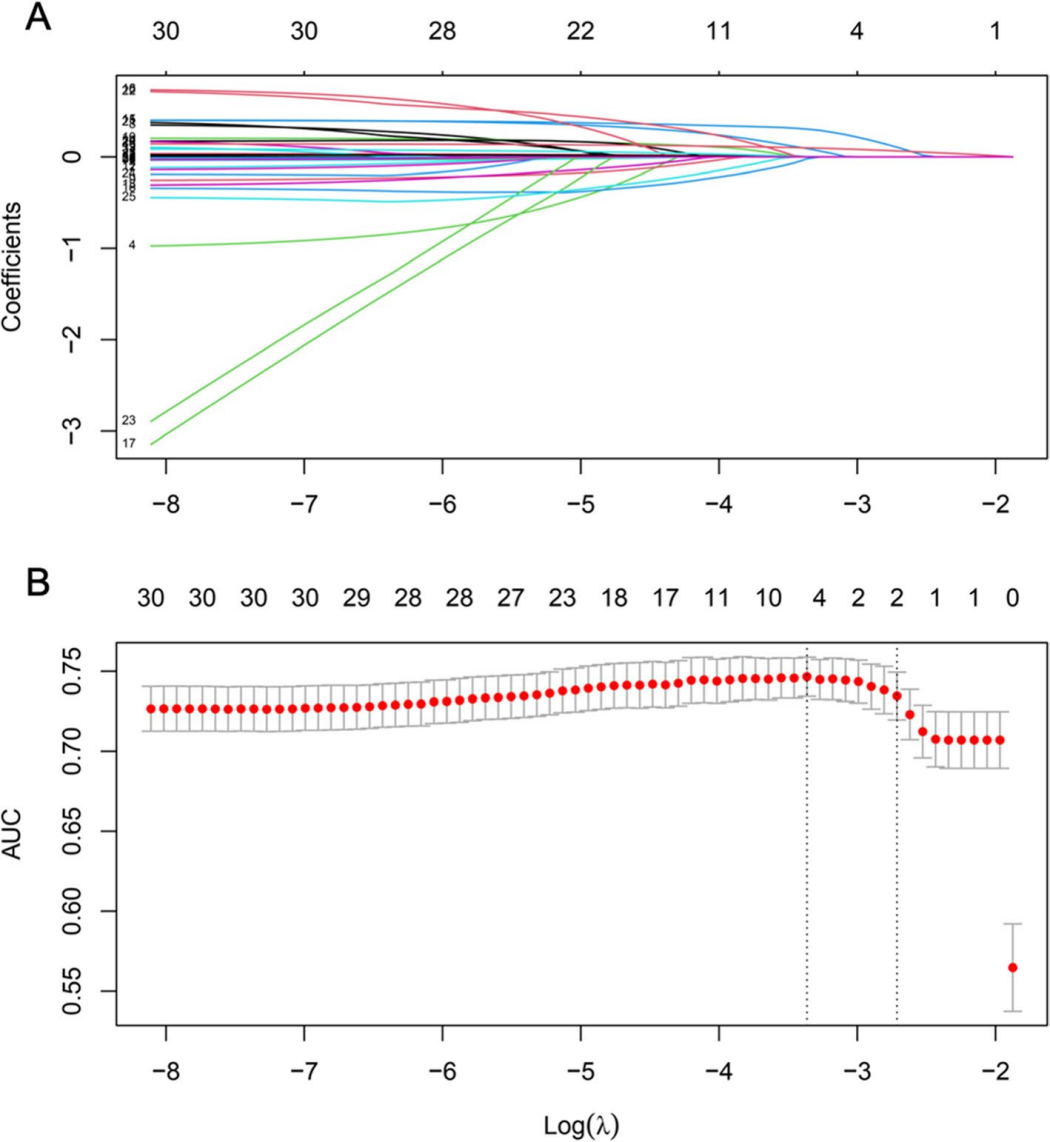
Feature selection using LASSO regression. **A** LASSO coefficient profiles of the clinical features. **B** Optimal penalization coefficient lambda (λ)

Thirteen variables measured at school (Tables 2 and 3) were included in the random forest. The process and results of feature selection by random forest are shown in Fig. 1, which identifies seven variables that were confirmed to be important: age, grade, employment status, father education level, game time, parental phubbing behavior, and general self-efficacy. Five variables were confirmed as unimportant: sex, mother’s education level, being an only child, number of people, and race. Furthermore, the variable of “household location” was excluded. To consolidate the results of the random forest feature selection, we performed a LASSO regression, as shown in Fig. 2. As expected, there were only two variables (grade and general self-efficacy) left in the LASSO regression model, far fewer than in the random forest model, due to the strong shrinkage capability of LASSO regression. These variables overlapped exactly with the variables identified in the random forest.

### Validation of CSEHL

**Fig. 3:**
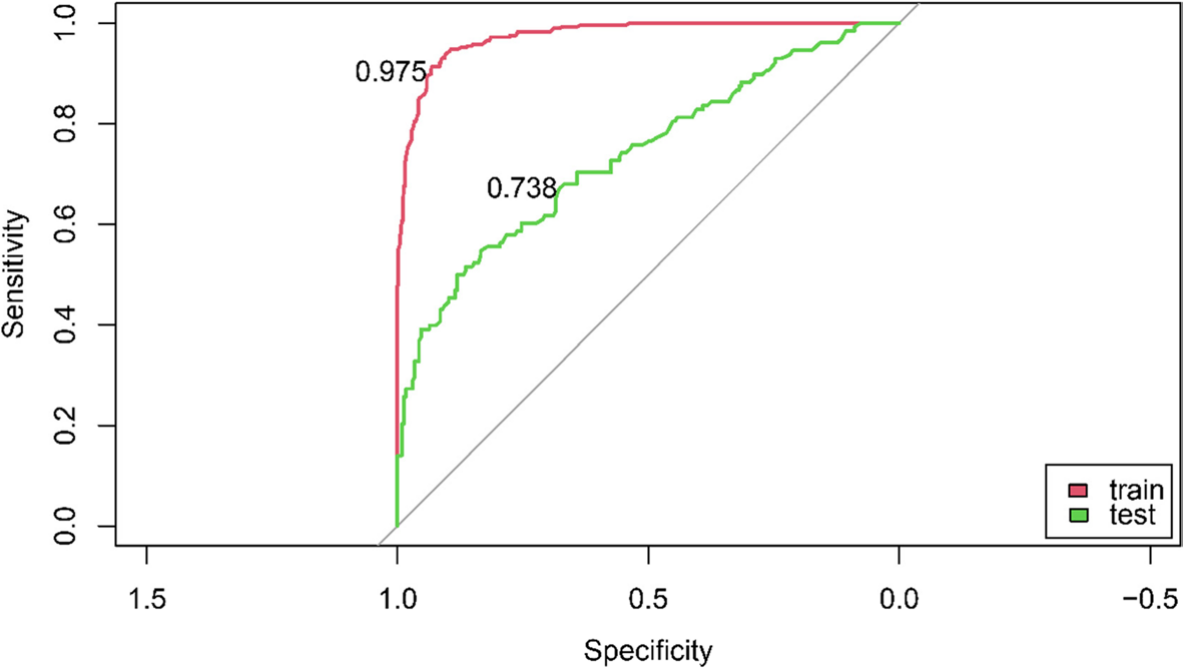
ROC for evaluating the model’s discrimination performance in the training and testing datasets. Red: Training dataset; a Green: Testing dataset

We then developed a prediction model using the seven identified key factors selected by the random forest. In the internal verification of the training dataset, the ROC showed that the model had high recognition ability, with an AUC of 0.975 (Fig. 3). The validation cohort included 363 students with a mean (SD) age of 13.5 (3.9) years, 188 (51.8%) males, and 128 (35.3%) students with EHL. In the independent validation database, the model showed satisfactory discrimination with an AUC of 0.738 (Fig. 3).

### Construction of the predictive score and web-based calculator

**Fig. 4:**
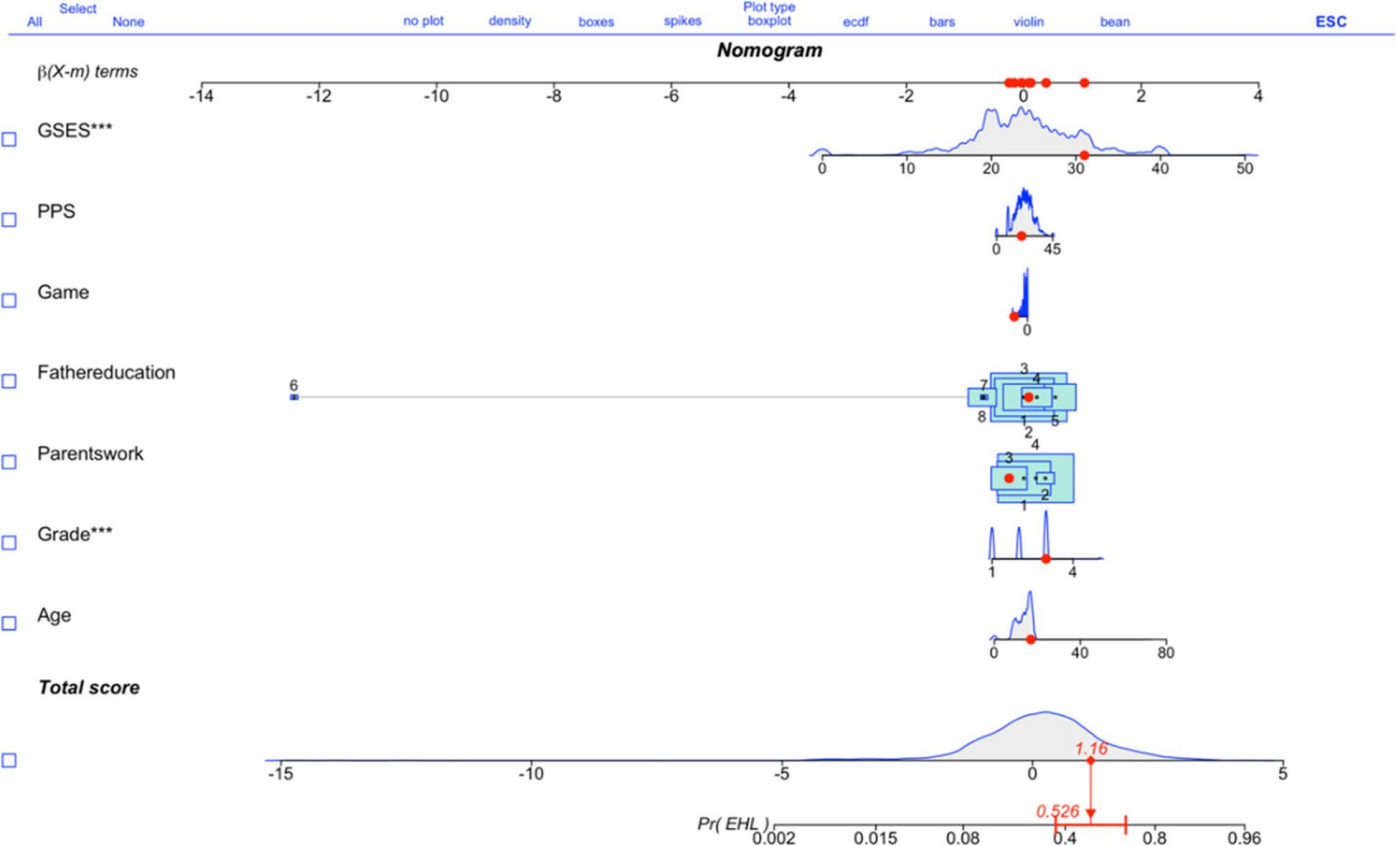
Nomogram of EHL

The EHL prediction score was constructed based on random forest. We used the model to build nomograms (Fig. 4). To further facilitate the use of our findings by policymakers and parents, this study presents nomograms in the form of a web page; that is, a web calculator was generated that can automatically calculate the probability of students having EHL according to seven key variables (https://xietao.shinyapps.io/DynNomapp/).

### Decision curve analysis

**Fig. 5:**
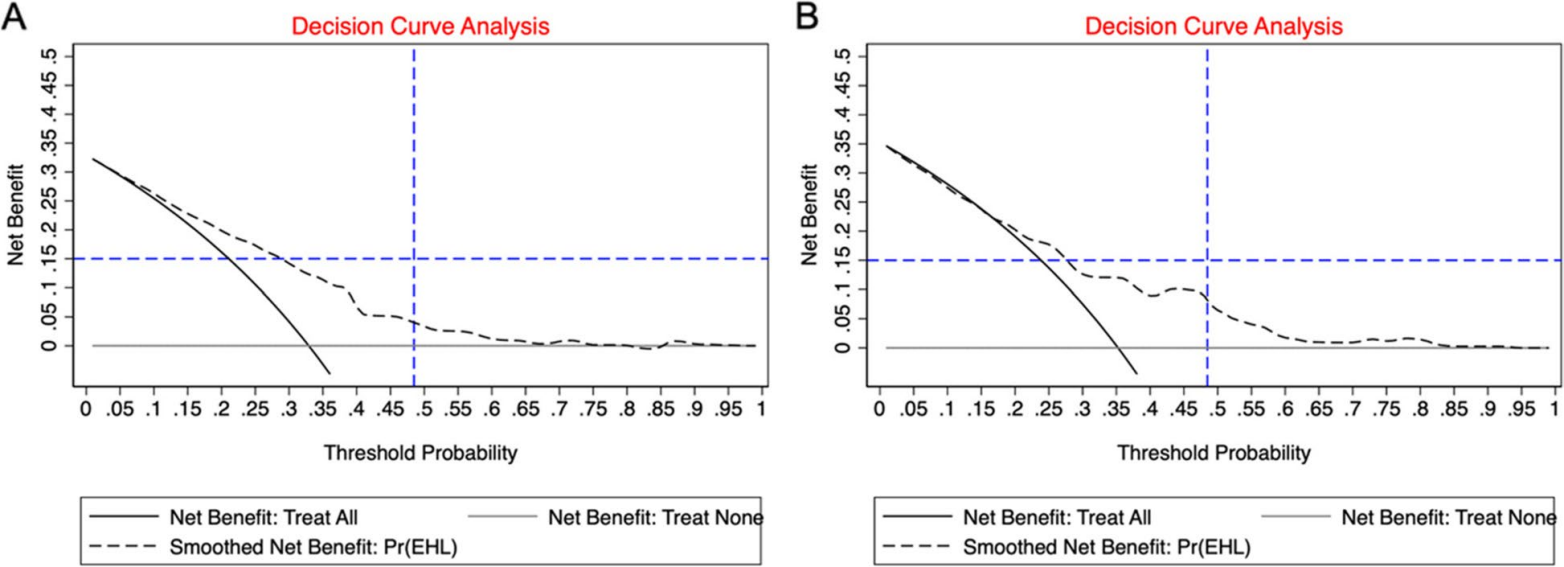
DCA focusing on the relationship between the benefits and risks brought by different models. Benefits and risks of models on training (**A**) /test (**B**) dataset

In Fig. 5, the three lines in the training and test parts represent the different conditions. Smoothed net benefit Pr (EH) represents the prediction model used in this study. The other two lines represent two extremes: net benefit: Treat none represents a situation where none of the samples have EHL, and the net benefit is zero. Net benefit: Treat indicates that all samples have EHL, and the net benefit is a negative slope of the backslash line. As shown in Fig. 5, the model in this study is higher than the extreme curve in a large threshold range. Therefore, the model in this study has a relatively large optional threshold range and is relatively safe. For example, in the training data set, assuming that we choose a prediction probability of 30%, 15 out of 100 students who use the model will benefit from it without affecting anyone else.

## Conclusions

Quality of access to health information is closely related to the quality of people’s lives. Knowing and processing health information and using it can help people maintain and promote their health. The internet is the main way to obtain health information [7]. An individual’s EHL will determine whether they can accurately obtain health information to promote their health. In this study, we developed and validated an EHL score map and a web-based web calculator to predict EHL among Chinese primary school students. In the training and validation datasets, the AUC values of the model were 0.975 and 0.738, respectively, which were satisfactory. Policymakers and parents can use our web-based calculator to estimate the probability of a student having EHL.

Mai et al. [34] pointed out that there were statistically significant differences in EHL scores among students of different sexes, places of household, and whether they were the only child. Multiple linear regression analysis found that the educational level of the father of a child was the main influencing factor of EHL. Zhong et al. [7] found that sex, grade, and time spent online were the main influencing factors of EHL in junior middle school students. We narrowed it down to seven key factors: age, grade, employment status, father’s education level, gaming time, parental phubbing behavior, and general self-efficacy. These factors are consistent with the results of previous studies.

Among the seven variables used to calculate the probability of EHL, age, grade, employment status, father’s education level, and game time can be obtained from the basic information. Basic efficacy and phubbing behavior can be measured using publicly available and easily available scales. Web-based calculators are easy to use, and schools and parents can take appropriate measures if it is identified that the probability of students having EHL is low. We have not graded the predicted probability so that parents of students in different regions can make decisions based on their family situation, and government workers can make decisions based on the development level of the region. For example, policymakers can intervene to help students whose predicted probability is below 80% in more developed provinces. However, in provinces with a general level of development, the prediction probability could be reduced to 60%. Different regions can explore the specific division of the prediction probability value themselves.

There are several limitations to this study. First, the sample size for constructing the probability score was moderate. Second, the sample size for verification was relatively small. Third, the sample size was concentrated in Shaanxi Province, China. These limitations may limit the applicability of the model to other regions of China. Data from other provinces in China must be collected to further verify the model. In addition, as mentioned above, because of the constraints of realistic conditions, this study did not include the variables of students’ academic performance and family income in the model, which needs to be overcome in future research.

## Supplementary Information

**Additional file 1. **

## Data Availability

All data generated or analyzed during this study are included in this published article.
