# Supplementary material for: How to predict the electronic health literacy of Chinese primary and secondary school students?: establishment of a model and web nomograms
Source: BMC Public Health. 2022 May 25;22:1048. doi: 10.1186/s12889-022-13421-4 (PMC9132355; doi:10.1186/s12889-022-13421-4)
Supplement: Supplementary file 1 — Additional file 1. [file 12889_2022_13421_MOESM1_ESM.docx]

Appendix 1

The internal consistency reliability and factor analysis results are presented in TableA 1. The Validity are presented in TableA 2. Each item in the eHEALS uses a 5-point Likert scale to answer each question with response options ranging from “strongly agree” to “strongly disagree”.

In this study, Cronbach's alpha coefficient and Construct Reliability (CR) were used to measure the Reliability of EHL, and the validity evaluation index was Average variance extraction amount (AVE). Item analysis was performed on the 8-items, producing a tight fitting scale with coefficient alpha of 0.9124. CR values were 0.9131. Both greater than 0.9. And through confirmatory factor analysis (CFA) (TableA 2). It indicates that the questionnaire has good internal consistency and high reliability. Principal components analysis was performed and produced a single factor solution as expected (eigenvalue = 4.970, 62.13% of the variance explained). Factor loadings ranged from 0.68 to 0.81 among the 8 items. The value of AVE is 0.57, indicating that this study item meets the criteria of convergence validity.

TableA 1 EHL of reliability and factor analysis

| Item | | Factor Loading |
| --- | --- | --- |
| 1 | I know how to find helpful health resources on the Internet | 0.6872 |
| 2 | I know how to use the Internet to answer my health questions | 0.7917 |
| 3 | I know what health resources are available on the Internet | 0.7600 |
| 4 | I know where to find helpful health resources on the Internet | 0.7811 |
| 5 | I know how to use the health information I find on the Internet to help me | 0.8058 |
| 6 | I have the skills I need to evaluate the health resources I find on the Internet | 0.7325 |
| 7 | I can tell high quality from low quality health resources on the Internet | 0.7796 |
| 8 | I feel confident in using information from the Internet to make health decisions | 0.6847 |
| Variance accounted for = 62.12%  Coefficient alpha = 0.9124 | | |

TableA 2 EHL of CFA

| Fitting index | | Validity index |
| --- | --- | --- |
| RMR = 0.045 | <0.05 | CR = 0.9131  >0.7 |
| RMSEA = 0.072 | <0.08 |  |
| AGFI = 0.949 | >0.9 |  |
| NFI = 0.982 | >0.9 | AVE = 0.57  >0.5 |
| GFI = 0.979 | >0.9 |  |
| IFI = 0.984 | 0.9 |  |
